# Metabolic reconstruction and experimental verification of glucose utilization in *Desulfurococcus amylolyticus* DSM 16532

**DOI:** 10.1007/s12223-018-0612-5

**Published:** 2018-05-24

**Authors:** Barbara Reischl, İpek Ergal, Simon K.-M. R. Rittmann

**Affiliations:** Archaea Physiology & Biotechnology Group, Archaea Biology and Ecogenomics Division, Department of Ecogenomics and Systems Biology, Universität Wien, Althanstraße 14, 1090 Wien, Austria

## Abstract

*Desulfurococcus amylolyticus* DSM 16532 is an anaerobic and hyperthermophilic crenarchaeon known to grow on a variety of different carbon sources, including monosaccharides and polysaccharides. Furthermore, *D. amylolyticus* is one of the few archaea that are known to be able to grow on cellulose. Here, we present the metabolic reconstruction of *D. amylolyticus*’ central carbon metabolism. Based on the published genome, the metabolic reconstruction was completed by integrating complementary information available from the KEGG, BRENDA, UniProt, NCBI, and PFAM databases, as well as from available literature. The genomic analysis of *D. amylolyticus* revealed genes for both the classical and the archaeal version of the Embden-Meyerhof pathway. The metabolic reconstruction highlighted gaps in carbon dioxide-fixation pathways. No complete carbon dioxide-fixation pathway such as the reductive citrate cycle or the dicarboxylate-4-hydroxybutyrate cycle could be identified. However, the metabolic reconstruction indicated that *D. amylolyticus* harbors all genes necessary for glucose metabolization. Closed batch experimental verification of glucose utilization by *D. amylolyticus* was performed in chemically defined medium. The findings from in silico analyses and from growth experiments are discussed with respect to physiological features of hyperthermophilic organisms.

**Electronic supplementary material:**

The online version of this article (10.1007/s12223-018-0612-5) contains supplementary material, which is available to authorized users.

## Introduction

Archaea were described as an independent phylogenetic group of microorganisms as early as 1977 (Woese and Fox 1977). The first two established archaeal phyla were the Euryarchaeota and the Crenarchaeota (Woese et al. 1990). Although archaea exhibit many prokaryotic characteristics, they also possess metabolic pathways that are markedly different from other organisms (Bräsen et al. 2014; Sato and Atomi 2011). The central carbohydrate metabolism of archaea comprises unique variants of enzymes within the Embden-Meyerhof-Parnas (EMP) pathway and the Entner-Doudoroff (ED) pathway (Bräsen et al. 2014; Verhees et al. 2003). The EMP pathway includes the phosphorylation of glucose or fructose to alpha-D-glucose-6-phosphate and fructose-6-phosphate, the cleavage of fructose-1,6-bisphosphate to glyceraldehyde-3-phosphate, and the further oxidation to glycerinaldehyde-3-phosphate by a glycerinaldehyde-3-phosphate:ferredoxin oxidoreductase (GAPOR). This is followed by phosphoglycerate mutase (PGM), an enolase, pyruvate kinase resulting in pyruvate, and the oxidation from pyruvate to acetyl-CoA by a pyruvate-ferredoxin oxidoreductase (PFOR). Anaerobic archaea possess the enzymes to convert acetyl-CoA to acetate by ADP-forming acetyl-CoA syntethase, while sulfur-, O_2_-, and nitrate-reducing archaea use the tricarboxylic acid cycle to oxidize it into two carbon dioxide (CO_2_) molecules (Siebers and Schönheit 2005). Based on the results of automated archaeal genome annotations, complete metabolic routes for known pathways could initially not be confirmed due to missing information on the archaeal enzymatic machinery. This was also the case when the CO_2_-fixing pathway was identified in Thaumarchaeota (Sato and Atomi 2011).

The ability to grow autotrophically is widespread among the Crenarchaeota and can be found among the orders Sulfolobales, Thermoproteales, and Desulfurococcales. Although no homologs of previously known and common CO_2_-fixation pathways have been found, enzymes for the 3-hydroxypropionate/4-hydroxybutyrate cycle or the dicarboxylate/4-hydroxybutyrate cycle have been identified, and their in vivo functionality has been experimentally verified (Bar-Even et al. 2011; Berg et al. 2010).

Among the Desulfurococcales, *Desulfurococcus amylolyticus* DSM 16532, formerly known as *Desulfurococcus fermentans* (Perevalova et al. 2016; Perevalova et al. 2005), was characterized as an anaerobic, hyperthermophilic crenarchaeon. *D. amylolyticus* was isolated from a freshwater hot spring in the Uzon caldera (Kamchatka Penninsula, Russian Federation). It is able to grow on a broad range of carbon sources including agarose, amygdalin, arabinose, arbutin, casein hydrolysate, dextran, dulcitol, fructose, lactose, laminarin, lichenan, maltose, pectin, peptone, ribose, starch, and sucrose. *D. amylolyticus* is known to possess metabolic and physiological differences compared to other *Desulfurococcus* spp.. While Desulfurococcaceae predominantly utilize either proteinaceous substrates or sugars (Perevalova et al. 2005; Susanti et al. 2012; Kublanov et al. 2009; Ravin et al. 2009), *D. amylolyticus* DSM 16532 possesses the ability to metabolize cellulose (Perevalova et al. 2005; Susanti et al. 2012). Also other archaea were shown to be able to metabolize cellulose, e.g., *Thermogladius calderae* (Kochetkova et al. 2016) and *Pyrococcus* spp. (Kishishita et al. 2015). Additionally, *D. amylolyticus* has the ability to tolerate 100% molecular hydrogen (H_2_) in the gas phase as well as using elemental sulfur as terminal electron acceptor. Moreover, sulfate and nitrate were shown not to influence growth (Perevalova et al. 2005; Kublanov et al. 2009). As mentioned before, most members of the Desulfurococcales grow heterotrophically, but recently, some members were shown to grow autotrophically by using the recently identified CO_2_-fixation pathways (Berg et al. 2010; Huber et al. 2008).

The aim of the presented work was to reassess, update, and comprehensively interpret the metabolic potential of *D. amylolyticus* with respect to selected mono-, di-, and polysaccharides. The in silico metabolic potential was examined including the genes for sugar transport and autotrophic growth. The genome annotation revealed that *D. amylolyticus* is able to metabolize glucose and harbors many enzymes that could enable CO_2_ fixation. Growth experiments were performed using different carbon compounds in closed batch cultivation mode. The intention was to reveal if *D. amylolyticus* would be able to grow on a chemically defined medium. In addition, physiological characteristics such as the specific growth rate (*μ*) and maximum cell concentration were determined during growth on different carbon substrates. Finally, we were able to verify that *D. amylolyticus* is able to grow on glucose.

## Material and methods

### Genome analysis

The genome of *D.* amylolyticus DSM 16532 (Z-1312) was sequenced by Susanti et al. 2012. The genome contains 1,384,116 bp with a GC content of 44.8%. One thousand seventy-five out of 1475 protein-coding genes were predicted to have known functions (Susanti et al. 2012).

Metabolic reconstruction of the *D. amylolyticus* genome was manually performed using the Kyoto Encyclopedia of Genes and Genomes (KEGG) (Kanehisa and Goto 2000), the Comprehensive Enzyme Information System (BRENDA) (Schomburg et al. 2002), the Universal Protein Resource (UniProt) (Boeckmann et al. 2005) domain database by the European Bioinformatics Institute (EMBL-EBI), and the Protein Family database (PFAM) (Finn et al. 2016). Enzyme information for the carbon metabolism (red), carbon fixation pathways in prokaryotes (red), glycolysis and glyconeogenesis (green), pyruvate metabolism (green), pentose phosphate pathway (orange), citrate cycle (yellow), starch and sucrose metabolism (blue), fructose and mannose metabolism (purple), galactose metabolism (gray), glyoxylate and dicarboxylate metabolism (pink), ABC transporter systems, and the secretion system were superimposed onto KEGG pathway maps (Supplementary Fig. 1).

NCBI database entries of gene-ID or protein-ID for each enzyme obtained from KEGG and BRENDA were analyzed using Basic Local Alignment Search (BLAST) (Altschul et al. 1990). BLAST was performed with the following settings: tblastn, tblastx, and blastn within the database reference genomic sequences (refseq_genomic) and blastp within the database reference proteins (refseq_protein). For a shorter calculation time, searches were limited to the taxa *Desulfurococcales* (taxid: 114380). Blastn calculations were extended to the BLAST algorithm “somewhat similar sequences.” Results for Query coverage, E-value, and Identity can be found in the supplemental material (Supplementary Tables 1 and 2). Supplementary Table 1 shows the complete list of enzymes, previously reported in KEGG enzyme maps, while Supplementary Table 2 shows enzymes of KEGG maps, which have not yet been assigned to the *D. amylolyticus* genome. The PFAM domain database was used to verify known protein family homologs based on the protein information received from BLAST results. All gene-IDs and protein-IDs shown in the proposed genome of *D. amylolyticus* have been checked for homologs PFAM families found in Supplementary Table 3. The identity threshold to determine homology was always ≥ 95%, except for PF00389 and PF02826 where the identity threshold was 70%.

### Chemicals

CO_2_, N_2_, 20 Vol.-% CO_2_ in N_2_, and CO were of test gas quality (Air Liquide, Schwechat, Austria). All other chemicals were of highest grade available.

### Microorganism and medium composition

*D. amylolyticus* DSM 16532 (Z-1312), formerly known as *D. fermentans* (Perevalova et al. 2016; Perevalova et al. 2005), was purchased from the Deutsche Sammlung von Mikroorganismen und Zellkulturen (DSMZ). A modified DSMZ medium No. 395 was used for all cultivations (per L): NH_4_Cl 0.33 g; KH_2_PO_4_ 0.33 g; KCl 0.33 g; CaCl_2_·2H_2_O 0.44 g; MgCl_2_·6H_2_O 0.70 g; NaCl 0.50 g; NaHCO_3_ 0.80 g; yeast extract (YE) 0.20 g; Na_2_S·9H_2_O 0.50 g; trace elements SL-10 1 mL; vitamin solution 10 mL. For cultures growing on a chemically defined medium, YE was excluded from the media solution. Carbohydrates (arabinose, fructose, glucose, lactose, maltose, starch, and sucrose) were supplied at a concentration of 5 g/L. Cellulose was tested at a concentration of 2 g/L, as at higher concentrations the cell densities could not be accurately determined. Experiments with CO_2_ (3·10^5^ Pa) and CO (2·10^5^ Pa) in the gas phase were performed using the aforementioned test gases. The trace elements solution (per L) was composed of: HCl (25 Vol.-%; 8.16 mol/L) 10 mL; FeCl_2_·H_2_O 1.50 g, ZnCl_2_ 0.07 g; MgCl_2_·4 H_2_O 0.1 g; H_3_BO_3_ 0.006 g; CoCl_2_·6H_2_O 0.19 g; CuCl_2_·2H_2_O 0.002 g; NiCl_2_·6H_2_O 0.024 g; Na_2_MoO_4_·2 H_2_O 0.036 g. Vitamin solution (per L): biotin 0.002 g; folic acid 0.002 g; pyridoxine-HCl 0.01 g; thiamine-HCl 0.005 g; riboflavin 0.005 g; nicotinic acid 0.005 g; d-Ca-pantothenate 0.005 g; vitamin B_12_ 0.0001 g; p-aminobenzoic acid 0.005 g; lipoic acid 0.0025 g. The medium was prepared anaerobically from individual solutions and then 50 mL of medium was distributed into 120-mL serum bottles with rubber stoppers (Butyl ruber 20 mm, Chemglass Life Science LLC, Vineland, USA). The serum bottle headspace consisted either of 20 Vol.-% CO_2_ in N_2_ or 100 Vol.-% N_2_. The serum bottle headspace was pressurized to 6·10^4^ Pa. The pH was subsequently adjusted to 6.2–6.4 with NaOH of appropriate molarity, using two different methods depending on the closed batch experiments (see below). Afterwards, the 120-mL flasks were sterilized at 121 °C. After sterilization, both vitamin solution and NaHCO_3_ solution were added separately inside a laminar-air-flow-chamber (FASTER BH-EN 2005, Szabo-Scandic, Vienna, Austria). Before inoculation, the medium was reduced by aseptically and anaerobically adding 0.4 mL of 0.5 mol/L Na_2_S·9H_2_O. To be able to cultivate *D. amylolyticus* on chemically defined medium, serial dilutions of fructose-grown *D. amylolyticus* were performed to allow the organism to adapt to a chemically defined medium lacking YE.

### Closed batch cultivation

Cultures of *D. amylolyticus* were grown anaerobically at 5·10^4^ Pa under either 20 Vol.-% CO_2_ in N_2_, 100% N_2,_ or 100% CO_2_ in a closed batch set-up (Rittmann and Herwig 2012). The following carbon sources were individually tested: arabinose, cellulose, fructose, glucose, lactose, maltose, starch, sucrose, CO, and CO_2_, with or without YE at 5 g/L, except for cellulose, which was applied at 2 g/L.

Two different approaches for the closed batch experiments were performed. In the closed batch experiments shown in Table 1**,** the pH was adjusted in each serum flask separately. The serum bottles were agitated at 100 rpm in an air bath (Labwit-Zwyr-2102c, Lab Xperts Laboratory Solutions Austria, Klosterneuburg, Austria). The pre-culture for inoculation was obtained from a fructose-grown *D. amylolyticus* culture. All experiments were performed in duplicates together with a negative control and reproduced three times. In the closed batch experiments shown in Table 2, the pH of the medium was adjusted in a 1-L gas-tight flask (pressure plus+ GL 45, clear, Duran Group, Mainz, Germany) before being distributed to the individual serum bottles sealed by rubber stoppers (Ochs Glasgerätebau, Langerwehe, Germany). The serum bottles were agitated at 200 rpm in an air bath (Labwit-Zwyr-2102c, Lab Xperts Laboratory Solutions Austria, Klosterneuburg, Austria). In the closed batch experiments shown in Table 2, fructose pre-grown *D. amylolyticus* cells were harvested by centrifugation (Eppendorf Centrifuge 5415R, Eppendorf, Hamburg, Germany) for 20 min and 15,700 g. The supernatant was removed and the resulting pellet washed with the respective medium. All experiments were performed in quadruplicates together with a negative control and reproduced twice. Pressure was always determined before samples for microscope analysis were obtained.

**Table 1:** Overview of closed batch experiments with YE supplementation

Substrate	*μ*_max_ [1/h]	*μ*_mean_ [1/h]	Final cell concentration [cells per mL]	Maximum doubling time [h]	Mean doubling time [h]
Starch	0.059	0.021	2.99·10^7^ ± 9.01·10^6^	12	33
Fructose	0.052	0.014	2.98·10^7^ ± 1.05·10^7^	13	50
Maltose	0.037	0.011	1.41·10^7^ ± 3.04·10^6^	19	63
Cellulose	0.021	0.011	1.41·10^7^ ± 4.49·10^6^	33	63
Arabinose	0.018	0.008	1.52·10^7^ ± 4.43·10^6^	39	87
Lactose	0.016	0.006	4.42·10^6^ ± 7.47·10^5^	43	116
Sucrose	0.009	0.004	4.91·10^6^ ± 1.02·10^6^	77	173

**Table 2:** Overview of closed batch experiments without YE supplementation

Substrate	*μ*_max_ [1/h]	*μ*_mean_ [1/h]	Final cell concentration [cells per mL]	Maximum doubling time [h]	Mean doubling time [h]
Cellulose	0.059	0.011	1.52·10^7^ ± 4.68·10^5^	12	63
Glucose	0.059	0.010	1.60·10^7^ ± 1.28·10^6^	12	69
Fructose	0.038	0.007	2.32·10^7^ ± 2.13·10^6^	18	99
CO_2_	0.004	0.003	2.73·10^4^ ± 2.73·10^2^	173	231
CO	0.002	0.001	2.80·10^5^ ± 1.01·10^5^	347	693

For inoculation, 5% (*v*/*v*) of pre-culture was added anaerobically in the anaerobic glove box (Coy Laboratory Products, Grass Lake, USA) using a gas-tight syringe (Soft-Ject, Henke Sass Wolf, Tuttlingen, Germany). After inoculation, the headspace of the bottles was filled with the respective gas and incubated at 80 °C in an air bath (Labwit-Zwyr-2102c, Lab Xperts Laboratory Solutions, Klosterneuburg, Austria). Depending on the specific growth rate (*μ*) of *D. amylolyticus* on different carbon substrates, samples of 1 mL of suspension were taken for cell counts at regular intervals. After sampling, the serum bottle headspace was re-prezurized to 5·10^4^ Pa using the respective gas. Cultivation in chemically defined medium was used to examine how *μ* and the final cell concentration of *D. amlyloyticus* are affected by the presence of the different carbon sources.

### Cell counting

Biomass samples were withdrawn from the serum bottles by using syringes (Soft-Ject, Henke Sass Wolf, Tuttlingen, Germany) and hypodermic needles (Sterican size 14, B. Braun, Melsungen, Germany). *D. amylolyticus* cells were counted by applying 10 μL of sample onto a Neubauer improved cell-counting chamber (Superior Marienfeld, Lauda-Königshofen, Germany) with a grid depth of 0.1 mm. Cultures were counted using a Nikon microscope (Nikon Eclipse 50i, Nikon, Amsterdam, Netherlands). Measurement of the absorbance for estimation of cell concentration could not be performed due to particle interference inside the cultivation media.

### Data analysis

The maximum specific growth rate (*μ*_max_ [1/h]) and the mean specific growth rate (*μ*_mean_ [1/h]) were calculated as follows: *N* = *N*_0_^.^*e*^μt^ with *N*, cell concentration [cells per mL]; *N*_0,_ initial cell concentration [cells per mL]; *t*, time [h]; and e, Euler number. *μ* was calculated from the delta cell counts (intervals) from the growth curves. *μ*_max_ is the maximum of the slope of all *μ*. *μ*_mean_ is the mean of all *μ* of the growth curve where an increase of cell concentration was visible.

## Results and discussion

### Substrate uptake

According to the metabolic reconstruction of *D. amylolyticus*, the organism is able to import a variety of carbon compounds by using a variety of genome encoded sugar transporters (Supplementary Fig. 1). These compounds include the monosaccharides glucose, fructose (hexoses) and arabinose (pentose), the disaccharides maltose, lactose and sucrose, and the polysaccharides starch and cellulose. This reconstruction differs from previous work in which glucose was shown not to be metabolized by *D. amylolyticus* (Perevalova et al. 2005). A complete list of all enzymes and PFAM domains can be found in Supplementary Tables 1, 2 and 3. *D. amylolyticus* DSM 16532 exhibited the highest gene similarity to *D. amylolyticus* 1221n (formerly known as *Desulfuroccus kamchatkensis*) and to *D. amylolyticus* Z-533^T^ (Supplementary Table 1**)**. This finding is not very surprising as *D. amylolyticus* DSM 16532 and *D. amylolyticus* 1221n were reclassified as synonyms of *D. amyloyticus* (Perevalova et al. 2016). According to our BLAST analyses, the carbon substrates could be channeled to the central catabolic pathway by 97 ABC transporter family genes, e.g., Desfe_0184, Desfe_0187, Desfe_0620, Desfe_0639, Desfe_0721, and Desfe_0754 as summarized in Supplementary Tables 1 and 2. However, the biochemistry of these ABC transporters is not known. Additionally, other enzymes in relation to sugar transport were identified in the genome, such as the multiple sugar transport system ATP-binding protein (Desfe_1188), which could channel the sugar via ABC transporter permease (Desfe_0355 and Desfe_0366) and the ABC transporter substrate-binding protein (Desfe_0354).

### Fermentative growth

**Fig. 1:**
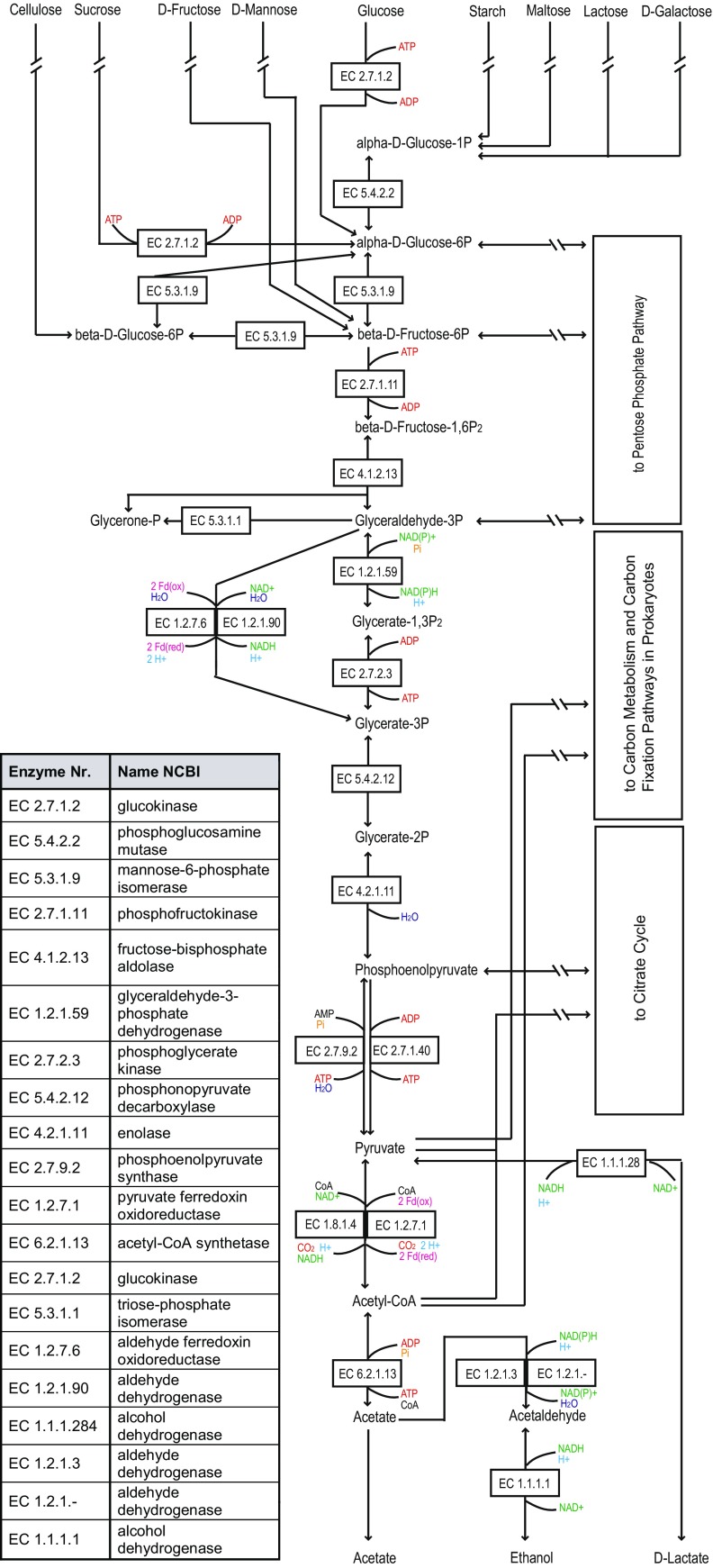
Predicted glycolysis and glyconeogenesis pathways and pyruvate metabolism of *D. amylolyticus* DSM 16532. (— // —): not all enzymes of the pathway are indicated. More detailed information on the carbon metabolism of *D. amylolyticus* DSM 16532 can be found in Supplementary Fig. 1

*D. amylolyticus* possesses all necessary genes for gluconeogenesis and glycolysis (Fig. 1). The glucose, fructose, and mannose degradation pathways lead to generation of beta-D-fructose-6-phosphate, where they enter glycolysis. This step could be followed by phosphofructokinase (PFK) (Desfe_0717 and Desfe_0968), fructose-bisphosphate aldolase, class I (Desfe_0718), and fructose 1,6-bisphosphate aldolase/phosphatase (FBPase, Desfe_1349). From results of the metabolic reconstruction, *D. amylolyticus* would be able to use the classical and the archaeal (modified) EMP to convert glyceraldehyde-3-phosphate to glycerate-3-phosphate. For the classical EMP, glyceraldehyde 3-phosphate dehydrogenase (GAPDH, Desfe_0262) and phosphoglycerate kinase (Desfe_0261) could be used, which would result in the production of NADPH and ATP. It must be noted that, except for halophilic archaea, GAPDH is involved in gluconeogenesis not in glyclolysis (Siebers and Schönheit 2005). However, in the archaeal EMP pathway, glyceraldehyde ferredoxin oxidoreductase (GAPOR, Desfe_0557) or a non-phosphorylating glyceraldehyde-3-phosphate dehydrogenase (GAPDH, Desfe_0067) could be used, which would result in 2 mol of reduced ferredoxin (Fd^2−^) or NADH respectively. FBPase (Desfe_1349) as well as GAPDH (Desfe_0067) are enzymes used in gluconeogenesis and counteract the irreversible reactions of the modified EMP, like PFK, GAPOR, and pyruvate kinase (Siebers and Schönheit 2005).

The archaeal EMP pathway was shown to have many variations, one of which has been shown to operate in *Pyrococcus furiosus* (Siebert and Schönheit 2005). Pyruvate, the end product of the EMP pathway (Kengen et al. 1996), was obtained via a 2,3-bisphosphoglycerate-independent phosphoglycerate mutase (Desfe_0416), enolase (Desfe_0063), pyruvate kinase (Desfe_1347), or pyruvate water dikinase (Desfe_0879). Pyruvate might be decarboxylated by PFOR to acetyl coenzyme A (acetyl-CoA). *D. amylolyticus* possesses 2 PFOR homologs: two alpha subunits (Desfe_0503 and Desfe_1298), two beta subunits (Desfe_0502 and Desfe_1299), two gamma subunits (Desfe_0505 and Desfe_1296), and two delta subunits (Desfe_0504 and Desfe_1297). This combined action would result in 2 Fd^2−^. *D. amylolyticus* possesses a dihydrolipoamide dehydrogenase (Desfe_0667) which could result in the production of 1 NADH through pyruvate degradation. However, the function of dihydrolipoamide dehydrogenase is only known in bacteria, where it catalyzes the oxidation of dihydrolipoamide. This reaction has not been shown in Archaea (Jolley et al. 1996). *D. amylolyticus* utilizes ATP-dependent sugar kinases during glycolysis (Kengen et al. 1994; Hansen and Schonheit 2000). Therefore, *D. amylolyticus* DSM 16532 must regenerate ATP for the anabolic reactions. Some of the ATP can be produced via substrate level phosphorylation through AMP-utilizing phosphoenolpyruvate synthase. Another possibility for ATP production could be chemiosmotic phosphorylation involving Fd^2−^ oxidation. Fd^2−^ can be produced through the action of GAPOR and/or PFOR. The oxidation of Fd^2−^ could be coupled to the generation of a proton motive force and subsequent production of H_2_ (Bräsen et al. 2014). *D. amylolyticus* possesses several homologs of hydrogenases, including a membrane-bound hydrogenase, which could be responsible for the generation of a proton motive force and concomitant H_2_ production. Despite very high cell-specific H_2_ production rates (3.41–8.42 fmol/cell h) were achieved during batch experiments in bioreactors, the volumetric H_2_ production rates of *D. amylolyticus* are still too low to be of biotechnological relevance (Reischl et al. 2018).

The metabolic routes for substrates to enter glycolysis differ for the different carbon compounds. With respect to monosaccharides, glycolysis may commence with the degradation of the monosaccharide glucose starting with a ROK family protein (Desfe_0578) to alpha-D-glucose-6-phosphate. From that point, the bifunctional phosphoglucose/phosphomannose isomerase (Desfe_1128) is used to generate beta-D-fructose 6-phosphate. These steps would consume 1 ATP. Fructose could enter via fructokinase (Defe_0717 and Desfe_0968) and may be converted to beta-D-fructose 6-phosphate while also consuming one ATP. Arabinose degradation in archaea is still unresolved and needs further investigation (Brouns et al. 2006). However, the gene annotations presented here indicate that *D. amylolyticus* possesses an alcohol dehydrogenase (Desfe_1240) that could form D-arabino-1,4-lactone and contains a D-arabino-1 dehydrogenase, which is a homolog to the dehydrogenase found in *Sulfolobus solfataricus* (SSO1300). Clusters of orthologous groups (COG) from various organisms for arabinose degradation such as COG3970, COG4948, COG0129, and COG0179 (Brouns et al. 2006) and genes for arabinose degradation found in *S. solfataricus*, e.g., SSO3124, SSO3117 and SSO3118 (Peng et al. 2011) or SSO3107, SSO1303 (Brouns et al. 2006), could not be detected in the genome of *D. amylolyticus*. Only a homolog of the 2-keto-3-deoxy-D-arabinonate dehydratase (COG3970), which is responsible for arabinose degradation, was found. Like in several *Burkholderia* species and in *Azospirillum brasiliense*, this gene could be replaced in *D. amylolyticus* by a dihydrodipicinolate synthase family protein (COG0329) (Brouns et al. 2006). When homologous genes from *Haloferax volcanii* are used for identification of genes for arabinose degradation in *D. amylolyticus*, only a gene encoding for a NAD-dependent epimerase (Desfe_0989) homologous to the *H. volcanii* gene HVO_B0032, which forms L-arabinoate, could be detected. Furthermore, no *H. volcanii* homologs for arabinose degradation (e.g. HVO_B0038A, HVO_B0027, or HVO_B0039 (Johnsen et al. 2013) could be identified in the genome of *D. amylolyticus*.

Concerning utilization of disaccharides by *D. amylolyticus*, sucrose could be split into D-fructose and alpha-D-glucose by a hypothetical protein, also annotated as sucrose alpha-glucosidase (Desfe_0611) and further transformed to alpha-D-glucose-phosphate by a ROK family protein (Desfe_0578) making use of 1 ATP. Lactose could be partitioned into D-glucose and D-galactose by beta-glucosidase (Desfe_0624) followed by a ROK family protein (Desfe_0578) generating alpha-D-glucose-6-phosphate. Maltose is split into 2 mol of glucose (Schäfer and Schönheit 1992) and could be degraded by maltokinase (Desfe_0406) using one ATP followed by the use of starch synthase (maltosyl-transferring, Desfe_0644) producing amylose and further metabolized to ADP-glucose by a starch synthase (glycosyl-transferring, Desfe_0403). With a sugar-phosphate nucleotidyltransferase (Desfe_0962) or via glucose-1-phosphate adenylyltransferase (Desfe_0189) that finally enters the glycolysis as alpha-D-glucose-1-phosphate, homologs of maltokinases (EC 2.7.1.175) are present in almost all known bacterial phyla as well as in some Crenachaeota (Fraga et al. 2015).

With respect to polysaccharide utilization, *D. amylolyticus* seems to possess several options. Starch could be broken down by starch phosphorylase (Desfe_0264) to amylose and alpha-D-glucose-1-phosphate. Amylose can be broken down by starch synthase (glycosyl-transferring) (Desfe_0403) to ADP-glucose. From ADP-glucose, glycolysis commences via nucleotidyltransferase (Desfe_0189) or sugar-phosphate nucleotidyltransferase (Desfe_ 0962) again forming alpha-D-glucose-1-phosphate. Cellulose could be degraded to cellobiose by an endoglucanase (Desfe_0691) and further to beta-D-glucose by a beta-glucosidase (Desfe_0624). Interestingly, no sequence homology to any known cellulose degrading enzyme could be found in the genome of *D. amylolyticus*. As our strain is unique among *Desulfurococcus* spp. to utilize cellulose (Graham et al. 2011), we compared the enzymes endoglucanase (Desfe_0691) and beta-galactosidase (Desfe_0624) to *D. amylolyticus* Z-533 T enzymes SPHMEL_RS03930 and SPHMEL_RS03240. There is a slight difference in the sequence: the endoglucanase gave an identity of 99% while the beta-galactosidase only showed 97% identity. Since it is known that the presence of cellulose genes does not assure the ability of an organism to be able to metabolize this substrate (Graham et al. 2011), the difference in gene identity was very interesting to note. Although the difference is not very high, the gene might be a new type of cellulase and the putative reason why the strain *D. amylolyicus* DSM 16532 is able to utilize cellulose. Hence, metabolomics and subsequent biochemical characterization seem to be necessary to elucidate the cellulose degradation pathway of this organism. Further degradation of cellobiose could be achieved by an ATP consuming step to beta-D-glucose-6-phosphate induced by a ROK family protein (Desfe_0578). Beta-D-glucose-6-phosphate then could enter glycolysis with the bifunctional phosphoglucose/phosphomannose isomerase (Desfe_1128) resulting in beta-D-fructose-6-phosphate.

The metabolic end product acetate could in theory be generated from acetyl-CoA by acetyl-CoA synthetase (Desfe_0782 and Desfe_1050), resulting in formation of 1 ATP. For synthesis of lactate and ethanol, reducing equivalents such as NAD(P)H and Fd^2−^ would have to be used. Aldehyde dehydrogenase (Desfe_0067) could catalyze the reaction from acetate to acetaldehyde, a very thermodynamic unfavorable reaction (Thauer et al. 1977), followed by alcohol dehydrogenase (Desfe_0019 and Desfe_1240) catalysis, which would form ethanol. Two NAD(P)H would be needed for these two reactions. The formation of the metabolic end product lactate would only require 1 NADH and could be catalyzed by a lactate dehydrogenase (Desfe_1212).

### Incomplete CO_2_-fixation pathways

Despite intensive examination and analysis of the *D. amylolyticus* genome, only an incomplete reductive citrate cycle could be identified, where certain essential genes are absent. To complete the reductive citric acid cycle in this organism, it would be necessary to find candidates for the missing enzymes in the genome and to perform biochemical characterization of the respective candidate enzymes. Unfortunately, this incomplete citric acid cycle is the case for many archaeal genomes (Vanwonterghem et al. 2016). *D. amylolyticus* could utilize the existing reactions of the reductive citric acid cycle for converting phosphoenolpyruvate (Desfe_0003) or pyruvate (Desfe_0591) to oxaloacetate. From there, oxaloacetate could be reduced to (S)-malate by malate dehydrogenase (Desfe_0284). The dehydration of malate to fumarate remains unresolved, as it is the case in *P. furiosus* and *Thermococcus kodakarensis*, although these two organisms are able to use a malic enzyme (EC 1.1.1.39) to dehydrate malate to pyruvate (Fukuda et al. 2005). However, no malic enzyme homologs could be identified in *D. amylolyticus*. Fumarate and succinate are interconverted by fumarate reductase/succinate dehydrogenase flavoprotein domain protein (Desfe_0481). Succinyl-CoA synthetase beta subunit (Desfe_1156) and succinyl-CoA synthetase alpha subunit (Desfe_1155) convert succinate to succinyl-CoA. The 2-oxoglutarate ferredoxin oxidoreductase complex including two alphas (Desfe_0475 and Desfe_0499), two betas (Desfe_0474 and Desfe_0498), one gamma (Desfe_0497), and one delta subunit (Desfe_0500) catalyze the reaction from succinyl-CoA to 2-oxoglutarate. Homologs for isocitrate dehydrogenase linking isocitrate to 2-oxoglutarate could not be identified in the genome. Citrate and isocitrate isomerization could be performed by aconitase (Desfe_0217). To be able to produce 2-oxoglutarate and aspartate, *D. amylolyticus* would be able to use aspartate aminotransferase (Desfe_0590) and further glutamate dehydrogenase (Desfe_0075) converting glutamate to 2-oxoglutarate while producing NADPH. Additionally, fumarate and aspartate utilization could be coupled via adenylosuccinate synthetase (Desfe_0482) and adenylosuccinate lyase (Desfe_0494).

The presence of other CO_2_-fixation pathways of Archaea, such as the dicarboxylate-4-hydroxybutyrate cycle, the 3-hydroxypropionate bicycle, and the 3-hydroxypropionate-4-hydroxybutyrate cycle, in the genome of *D. amylolyticus* were also investigated (Supplementary Table 4). However, despite intensive in silico examinations, no complete CO_2_-fixation pathway (Bar-Even et al. 2011; Berg et al. 2007; Berg et al. 2010; Huber et al. 2008; Thauer 2007) could be identified. Within the dicarboxylate-4-hydroxybutyrate cycle (Berg et al. 2010), only the conversion from succinate to succinyl-CoA (Desfe_1155 and Desfe_1156), the oxidation to succinate semialdehyde (Desfe_0067) and the transformation to 4-hydroxybutyrate by homologs of alcohol dehydrogenase (Desfe_1240 and Desfe_0019), and the subsequent transformation from acetoacetyl-CoA to two acetyl-CoA (Desfe_0849) could be proposed to take place in *D. amylolyticus*. The other steps necessary for performing the dicarboxylate-4-hydroxybutyrate cycle (Bar-Even et al. 2011; Huber et al. 2008) remain undetected. Similarly, only a few genes encoding reactions of the 3-hydroxypropionate bicycle (Bar-Even et al. 2011; Berg et al. 2010) were found in the genome, including the enzyme for formation of acrylyl-CoA from 3-hydroxypropionate (Desfe_0019 and Desfe_1240) and the enzyme conversion of acrylyl-CoA to propionyl-CoA (Desfe_0880).

Our extensive genome re-annotation revealed that *D. amylolyticus* harbors several genes involved in various CO_2_ fixation pathways, but from a metabolic reconstruction perspective, this organism seems to rely on the fermentative growth mode for maintaining its carbon and energy metabolism.

### Determination of physiological characteristics

Based on the metabolic reconstruction, closed batch experiments were conducted to determine the carbon source resulting in the highest *μ*_max_, *μ*_mean_, and highest final cell densities. Therefore, *D. amylolyticus* was grown on selected carbon compounds (arabinose, cellulose, fructose, glucose, lactose, maltose, sucrose, and starch) and on CO_2_ and CO in a closed batch system.

**Fig. 2:**
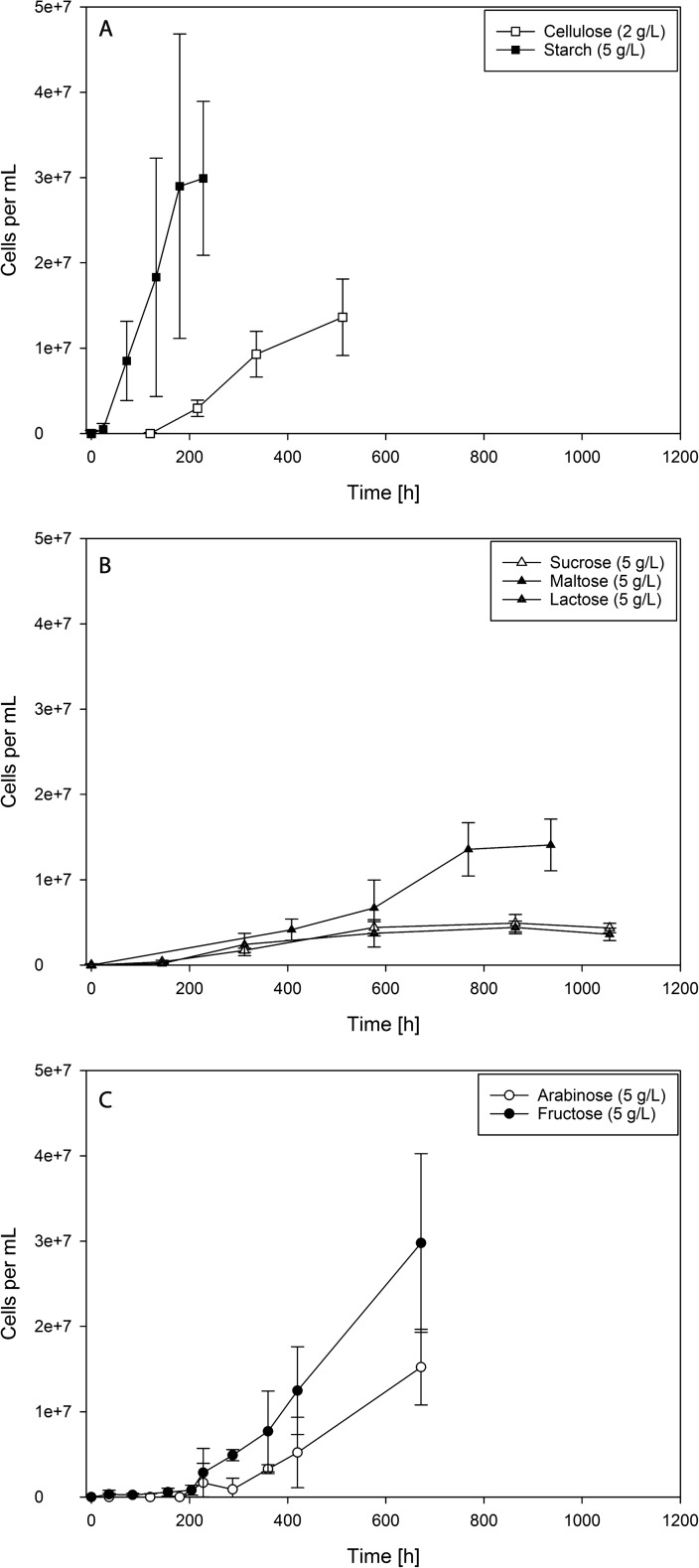
Growth curves of *D. amylolyticus* from closed batch experiments with YE supplementation. **a** Growth of *D. amylolyticus* on polysaccharides. **b** Growth of *D. amylolyticus* on disaccharides. **c** Growth of *D. amylolyticus* on monosaccharides

*D. amylolyticus* was able to grow on all tested poly-, di-, and monosaccharides. Growth curves of *D. amylolyticus* grown in a medium containing one of these sugars and YE is shown in Fig. 2. Unambiguously, growth on starch (Fig. 2a, Table 1), provided the best growth conditions resulting in a *μ*_max_ of 0.059 1/h and a cell concentration of 3.00·10^7^ confirming earlier reports (Perevalova et al. 2005). When considering the metabolic burden of sugar transport into the cell to produce cellular energy, the constraints of the fermentative growth of *D. amylolyticus* on starch, accompanied by acetate formation, could result in the best growth conditions and highest cellular energy gain. However, during the cultivation of *D. amylolyticus* on starch solid particles, aggregation occurred inside the growth medium which made cell counting difficult to almost impossible (as indicated in the standard deviation shown in Fig. 2a). Slow growth and growth to low cell densities could be observed when *D. amylolyticus* was grown on disaccharides (Fig. 2b and Table 1). This could be explained by putative unintended by-product formation and accumulation due to the Maillard reaction (Lerche et al. 2002), which was inhibiting growth of *D. amlyolyticus*. According to a study on anaerobic bacteria, melanoidins, the product of the Maillard reaction, have strong prebiotic potential and can be used as carbon source by particularly *Bifidobacterium* spp. (Borrelli and Fogliano 2005). However, the effect on archaea could be different. Another reason could be that trace elements (e.g., tungsten and/or selenium) were limiting growth. Possibly also the sulfur source for growth of *D. amylolyticus* could be reconsidered, as sulfide is known to precipitate metal ions. As an alternative, growth tests using cysteine instead of sulfide could be performed. However, the generally encountered characteristic of hyperthermophilic organisms to grow only to low cell densities could be circumvented by applying cell retention systems, or by using multivariate optimization procedures for improving final biomass concentration values, as they have already been employed for thermophilic microorganisms (Abdel Azim et al. 2017; Seifert et al. 2014). Yet, there are still many unknown parameters that limit growth of hyperthermophiles to high cell densities (Chou et al. 2008; Pawar and Niel 2013). The fastest growth of *D. amylolyticus* on monosaccharides and YE was obtained from fructose (Fig. 2c). Growth of *D. amylolyticus* on fructose comprised a *μ*_max_ of 0.052 1/h and reached a cell concentration of 2.98·10^7^.

**Fig. 3:**
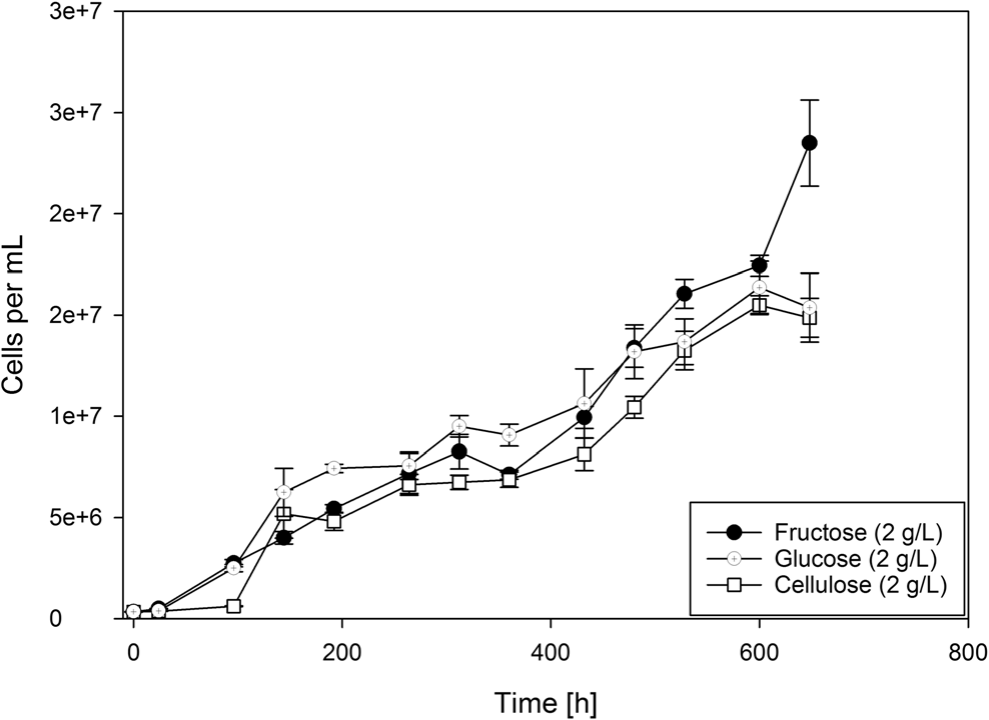
Growth curves of *D. amylolyticus* from closed batch experiments in defined medium on cellulose, fructose, and glucose

From our metabolic reconstruction, there was no convincing indication that *D. amylolyticus* would not be able to grow on glucose (Fig. 1). Therefore, growth of *D. amylolyticus* was tested in a chemically defined medium containing fructose, cellulose, or glucose (Fig. 3). In contrast to Perevalova et al. 2005 (Perevalova et al. 2005), growth of *D. amlylolyticus* on glucose was achieved at a *μ*_max_ of 0.059 1/h. Even though the metabolic reconstruction revealed enzymes that could be involved in CO_2_ fixation, almost no growth could be detected when CO_2_ was used as sole source of carbon for growth (Table 2). This is not surprising and might be due to the fact that in addition to CO_2_, another energy source, e.g., H_2_ would be needed for cultivation of *D. amylolyticus*. Also, almost no growth could be observed when *D. amylolyticus* was grown on CO (Table 2). In the latter experimental setting, *D. amylolyticus* was always grown in a chemically defined medium lacking YE (Fig. 3). However, the addition of YE stimulated growth of *D. amylolyticus* (compare individual growth curves of Figs. 2 and 3). YE is very expensive and a source of rich complex nutrients, proteins, and minerals and therefore aimed to be omitted if a biotechnological production processes is envisioned (Willquist and van Niel 2012). Furthermore, the omission of YE is a prerequisite for physiological studies which aim to achieve fine-resolution mass balancing analyses.

According to the metabolic reconstruction of *D. amylolyticus* (Supplementary Fig. 1), growth on all tested carbohydrates would result in standard glycolytic ATP gain or loss. Therefore, the future research question would be how and if *D. amylolyticus* is able to gain and maintain redox equivalent homeostasis, or if cellular energy can also be obtained from co-assimilation of YE. As in their natural environment archaea could encounter carbon oligotrophic conditions, the organism could be growing mixotrophically. A putative co-assimilation of certain components contained in YE might be advantageous for *D. amylolyticus*. In this respect, also a rudimentary reverse citric acid cycle or a rudimentary 3-hydroxypropionate/4-hydroxybutyrate cycle could assist the balance of anaplerotic and cataplerotic reactions (Berg et al. 2010). An option could be to use carbon isotope labelling studies for elucidation of the CO_2_ fixation potential or gene expression analysis. Another discussion point concerns the affinity of ABC transporters towards certain sugars (Bräsen et al. 2014; Willquist et al. 2010). The determination of ABC transporter specificity could be beneficial in designing future experiments designed to understand under which growth conditions and from which carbohydrate the highest biomass concentrations could be obtained. Such a physiological understanding would be necessary in order to achieve high biomass concentrations for subsequent biochemical, physiological, and biotechnological studies.

## Electronic supplementary material

Supplementary Figure 1 Predicted metabolism of *D. amylolyticus* DSM 16532 with information about the carbon metabolism (red), carbon fixation pathways in prokaryotes (red), glycolysis and glyconeogenesis (green), pyruvate metabolism (green), pentose phosphate pathway (orange), citrate cycle (yellow), starch and sucrose metabolism (blue), fructose and mannose metabolism (purple), galactose metabolism (gray), glyoxylate and dicarboxylate metabolism (pink), ABC transporter systems, and the secretion system. No homologs could be found on enzyme numbers filled in gray. (PDF 541 kb)

Supplementary Table 1 The complete list of enzymes of *D. amylolyticus*, previously reported in KEGG enzyme maps. (XLSX 60 kb)

Supplementary Table 2 Enzymes of KEGG maps, which have not yet been assigned to *D. amylolyticus* genome. (XLSX 86 kb)

Supplementary Table 3 Gene-IDs and protein-IDs shown in the proposed genome of *D. amylolyticus* have been checked for homologs PFAM families. Red mark indicates that no homolog PFAM has been found. (XLSX 58 kb)

Supplementary Table 4 Search for homologs in proposed CO_2_-fixation pathways of archaea: the dicarboxylate-4-hydroxybutyrate cycle, the 3-hydroxypropionate bicycle, and the 3-hydroxypropionate-4-hydroxybutyrate cycle. (XLSX 18 kb)
