# Supplementary figures and images for: Metabolic reconstruction and experimental verification of glucose utilization in Desulfurococcus amylolyticus DSM 16532
Source: Folia Microbiol (Praha). 2018 May 24;63(6):713–23. doi: 10.1007/s12223-018-0612-5 (PMC6182646; doi:10.1007/s12223-018-0612-5)

### Predicted Metabolism of *Desulfurococcus amylolyticus*

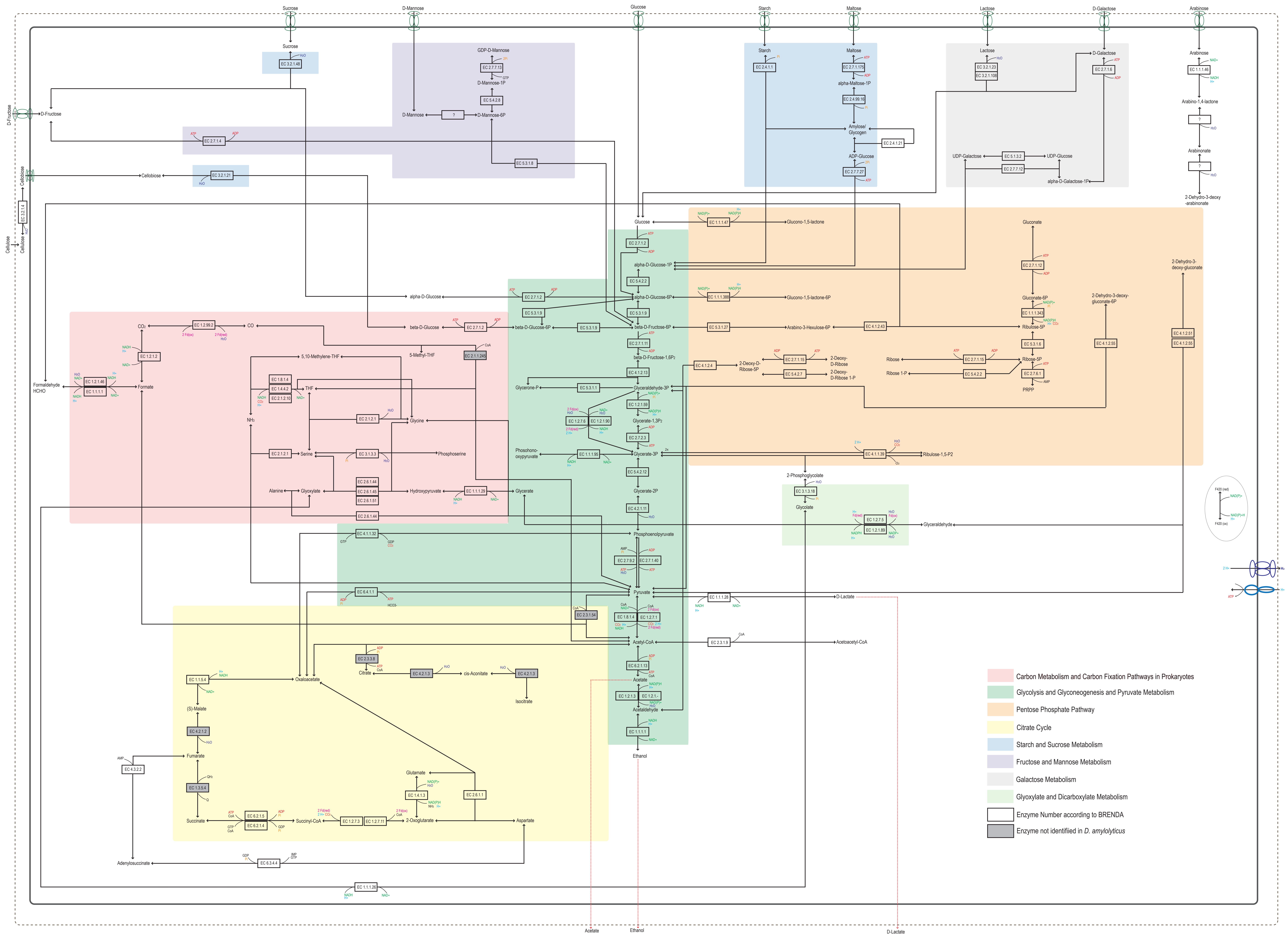

Supplement: Supplementary file 1 — Predicted metabolism of D. amylolyticus DSM 16532 with information about the carbon metabolism (red), carbon fixation pathways in prokaryotes (red), glycolysis and glyconeogenesis (green), pyruvate metabolism (green), pentose phosphate pathway (orange), citrate cycle (yellow), starch and sucrose metabolism (blue), fructose and mannose metabolism (purple), galactose metabolism (gray), glyoxylate and dicarboxylate metabolism (pink), ABC transporter systems, and the secretion system. No homologs could be found on enzyme numbers filled in gray. (PDF 541 kb) [file 12223_2018_612_MOESM1_ESM.pdf]
